# ToxPoint: Copper Is the New Showstopper

**DOI:** 10.1093/toxsci/kfac071

**Published:** 2022-08-19

**Authors:** Govind Gupta, Bengt Fadeel

**Affiliations:** Institute of Environmental Medicine, Karolinska Institutet, 171 77 Stockholm, Sweden; Institute of Environmental Medicine, Karolinska Institutet, 171 77 Stockholm, Sweden

**Keywords:** copper, cuproptosis, mitochondria, nanotoxicology, trace element

Copper is an essential trace element found in all living organisms in its oxidized Cu(II) and reduced Cu(I) states. Indeed, the inherent redox properties of copper make it beneficial as well as potentially deleterious. Therefore, cellular copper homeostasis is subject to strict control. Previous studies have implicated mitochondria in copper-induced toxicity but exactly how copper kills cells remained unknown. Similarly, the exact mechanism whereby copper-based nanoparticles trigger cellular or organismal toxicity has remained unclear. However, it is worth noting that the toxicology of nanoparticles is, to a large extent, the toxicology of metals and metal oxides; indeed, even nonmetallic (carbonaceous) nanomaterials may contain residual transition metals.

In a recent landmark study, the mechanism of copper-induced cell death has been clarified. Using elesclomol, a so-called copper ionophore, Tsvetkov *et al.* (2022) demonstrated that copper triggers a nonapoptotic form of cell death that relies on mitochondrial respiration. Elesclomol forms a 1:1 complex with Cu(II) and is capable of transporting Cu(II) from the extracellular environment into mitochondria where it is reduced to Cu(I). The authors could show in a previous study that the iron-sulfur protein ferredoxin 1 (FDX1) is responsible for the reduction of elesclomol-bound Cu(II) to Cu(I) in mitochondria, leading to susceptibility to copper-induced cell death in certain cancer cells (Tsvetkov *et al.*, 2019). Indeed, elevated *FDX1* gene expression was identified as a robust biomarker for elesclomol-induced toxicity across a panel of more than 700 cancer cell lines (Tsvetkov *et al.*, 2019). Interestingly, the related mitochondrial ferredoxin, FDX2, and its yeast homolog, Yah1, were previously shown by other investigators to determine cellular vulnerability to copper (Vallières *et al.*, 2017). Thus, the mitochondrial ferredoxins have emerged as important and conserved targets of cellular copper excess. Indeed, these iron-sulfur proteins may be viewed as an Achilles’ heel with respect to cellular vulnerability to excess copper or as (novel) regulators of copper-induced cell death in cancer cells, or both. Tsvetkov *et al.* (2022) also unearthed a role for protein lipoylation, an unusual but highly conserved posttranslational modification, in the modulation of copper-induced cell death. Notably, lipoylation and the cellular machinery that is required to add or remove lipoic acid, and the lipoylated (mitochondrial) target proteins, are all highly conserved. Tsvetkov *et al.* (2022) found that copper toxicity could be suppressed by genetic disruption of the genes required for lipoylation or of the lipoylated enzymes themselves. The fact that lipoylated proteins in mitochondria act as a sink for copper should not come as a surprise as previous work has shown that the reduced form of lipoic acid displays substantial Cu(I)-binding affinity (Smirnova *et al.*, 2018), higher than that of glutathione but lower than the cysteine-rich, metal-binding metallothioneins (Banci *et al.*, 2010). Tsvetkov *et al.* (2022) also found that copper caused aggregation of lipoylated proteins, leading to cell death, and they could show that this cell death was distinct from the known cell death modalities, apoptosis, necroptosis, and ferroptosis. Indeed, it is well known that caspase-3, the main executioner of apoptosis, is susceptible to inactivation through thiol oxidation, and this may explain why the apoptosis machinery is incapacitated in cuproptosis. Hence, it can be speculated that copper ionophores may be useful for the treatment of cancers that are apoptosis resistant, provided that normal cells are protected from the toxicity of excess copper. It was previously shown that the mitochondrial sirtuin, SIRT4, acts as a cellular lipoamidase to remove lipoyl modifications (Mathias *et al.*, 2014), and it is conceivable that SIRT4 could modulate the toxicity of excess mitochondrial copper, though this remains to be tested.

Do these results have any bearing on the toxicity of nanoparticles, or, conversely, can nanotoxicology shed any light on the mechanisms of action of excess cellular copper? Cytosolic Cu, Zn-SOD1 is known to have one of the highest affinities for Cu(I) (Banci *et al.*, 2010). SOD1 is ubiquitous in mammalian cells, and it has been suggested that aerobic life depends principally on the superoxide dismutases and their ability to scavenge superoxide radicals. Using mouse macrophages as a model, copper oxide nanoparticles were recently found to be internalized by cells and to undergo dissolution leading to a cellular copper overload with misfolding of SOD1 (Gupta *et al.*, 2022). Cells exposed to the copper oxide nanoparticles displayed oxidative stress with swelling of mitochondria (a sign of necrosis), and cell death was found to be nonapoptotic, thus disproving the common assumption that copper triggers apoptosis. In fact, it seems plausible that the cellular “delivery” of copper, whether through copper ionophores (Tsvetkov *et al.*, 2022), or through the uptake of copper-based nanoparticles (Gupta *et al.*, 2022), triggers a common form of copper-dependent cell death.

Of course, a major difference between the use of copper ionophores such as elesclomol and nanoparticles is that the source of copper in the case of ionophore-treated cells is the serum present in the cell culture medium while in the case of nanoparticle exposure, a massive amount of copper, by comparison, is internalized by cells. The dose makes the poison, and for copper, it is the intracellular dose that matters.

Moreover, copper-based nanoparticles were found to trigger protein aggregation and proteasomal inhibition, in line with previous transcriptomics and proteomics studies showing that copper nanoparticles elicit an unfolded protein response (Gupta *et al.*, 2022). Intriguingly, another recent study in which the toxicity of copper toward the bacterium *Escherichia* *coli* was explored further supported a role of copper-induced protein aggregation in the cytotoxicity of copper (Zuily *et al.*, 2022). The authors found that bacteria lacking the cytosolic chaperone DnaK (the bacterial homolog of HSP70) were susceptible to copper stress. Thus, proteotoxic stress is another common feature of copper-induced toxicity, and it seems that heat shock proteins may protect against excess copper. In addition, previous work has shown that hepatocytes can regulate the levels of copper through the exocytosis of lysosomes. It will be important to understand whether copper-based nanoparticles could also be expelled through exocytosis, as such nanoparticles tend to congregate in lysosomes (Gupta *et al.*, 2022).

Taken together, the recent flurry of investigations on copper has uncovered how an essential element can become toxic. They also suggest that the intersection between cell biology and (nano)toxicology may be fertile ground for future studies of (toxic) metals. The new findings regarding copper-mediated protein aggregation and mitochondrial stress extend beyond the role of copper as a potential cancer therapeutic and may shed light on neurodegenerative diseases as well as liver diseases such as Wilson disease, an autosomal recessive disorder of copper deposition caused by mutations in the copper-transporting ATPase, *ATP7B*. Moreover, cuproptosis may also be relevant for toxicities caused by occupational or environmental exposures to copper.
